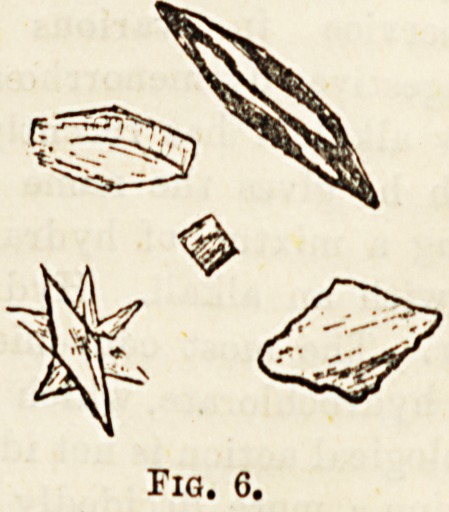# The Microscope in Medicine

**Published:** 1891-04-04

**Authors:** Frank J. Wethered


### The Microscope in Medicine.

By Frank J. Wethered, M.D.

### XI.—THE EXAMINATION OF URINARY DEPOSITS.

*(Continued.)*

[Two illustrations were inadvertently used in the last article which did not refer to the subject matter.]

Casts.—These bodies, when present, are subject to very great variety as to their number, their shape, and the conclusions which may be drawn from their presence. They have been evidently moulded in the renal tubules. Their basis is apparently an albuminous material exuded from the capillaries, or derived from the colloid degeneration of epithelial cells. Though in the vast majority of cases indicative of a morbid condition of the kidneys, they are occasionally found in the absence of albumen. Thus, Nothnagel has seen them in the urine of patients suffering from jaundice, and other observers have described them in cases of acute inflammation of the stomach and intestine.

The method for searching for them differs in no way from those already described in treating of the other urinary deposits. Care must be taken not to confuse linear collections of urates for casts. When the deposit of lithates is very copious, and a small portion placed on a slide and protected by a cover-glass, small groups are often seen which very closely resemble the real cylinders. The former may, however, be distinguished from the latter by disappearing when the cover-glass is slightly moved.

The most common form is the *hyaline* (Fig. 2). They are colourless delicate bodies of varying length and thickness, and bounded by faintly marked outlines. On account of their low refracting power they are not easily detected unless some staining solution has been employed. Attention is often

Fig. 2.

drawn to them by cells and crystals lying on them. Their pathological significance is not very great. They are most constantly met with in chronic Bright's disease, but have also been found in urine which was apparently healthy (Henle).

Closely similating these are the *amyloid* casts. In appearance they cannot be distinguished from the hyaline, but may be known by the peculiar brown stain given to them by a solution of iodine. They occur in all forms of renal disease, acute and chronic, and in large numbers in amyloid disease of the kidney.

*Granular* casts (Fig. 3).—These are the most common. They vary much in size, and are usually seen as fragments. Their granular consistence is changeable. Sometimes the particles are so fine that they can only be recognised under a high power. At other times the granules are coarser. They are opaque, and their colour may be any shade from grey to

Fig. 3.

reddish brown. One characteristic is their sharp contour. They are of more diagnostic importance than the two forms just described, for they always indicate a chronic inflammatory state of the kidneys. They may occur together with blood casts, in which case it is proved that an acute nephritis has supervened upon a chronic one.

*Epithelial* casts (Fig. 4).—As their name indicates, these consist of immature forms of epithelium embedded in the

Fig. 4.

hyaline albuminoid substance. The individual cells are usually circular in shape, more rarely polygonal. They denote inflammatory condition of the kidneys.

*Pus* casts.—These are very rarely met with. They are usually associated with the discharge of minute abscesses into the tubules.

*Blood* casts (Fig. 5).—These are easily recognised by their colour and uniformity in size of the cells forming them. They are always indicative of some acute condition, generally a nephritis, but also accompany an intense congestion such

Fig. 5.

as results from the administration of turpentine or cantharides. Occasionally also after injuries of the kidney the urine contains these casts, and the same may be said when a renal calculus is present. Finally, they have been described in cases of tubercle, infarct, and new growth.

*Fat* casts.—These are also uncommon. Fatty globules are, indeed, not unfrequently seen upon the surface of granular casts, but occurring in such large numbers as to form distinct cylinders is a rare condition. In some instances they are covered with needles of fatty crystals. When found in this condition they indicate an advanced state of fatty degeneration of the kidney. The only other form of cast met with are those composed of *colonies of micrococci*. They are very similar in appearance to the granular casts just described, but may be distinguished from them by their resistance to reagents, such as caustic potash and nitric acid. They make the prognosis very grave, implying as a rule septic embolism of the kidney, or pyelo-nephritis.

Having thus disposed of the organised urinary sediments, we now pass to the non-organised portion. In order to collect them the urine is allowed to stand twenty-four hours, as stated previously, the supernatent fluid poured off, and portions removed to slides by means of a pipette. The sediments vary according as to whether the urine is alkaline or acid. We will consider the *deposits* occurring in *acid urine* first.

*Uric acid* (Fig. 6)—Pure crystalline uric acid is colourless, but when deposited from urine it is more or less deeply stained a brownish yellow colour, the tint depending upon the amount of colouring matters in the secretion. The

Fig. 6.

crystals differ very much in form and size. For the most part they consist of rhomboidal plates which are frequently aggregated together into stellate masses, but often assume a rounded, barrel-like, or dumb-bell form. When seen at the bottom of the collecting glass, they have very much the appearance of the grains of cayenne pepper. Uric acid occurs in various conditions, the most important of which are gout, rheumatism, febrile conditions, and chroni diseases of the heart and lungs.

*Phosphates.*—These are more commonly seen in alkaline urine, and will be described under that head.

*(To be continued.)*

## Figures and Tables

**Fig. 2. f1:**
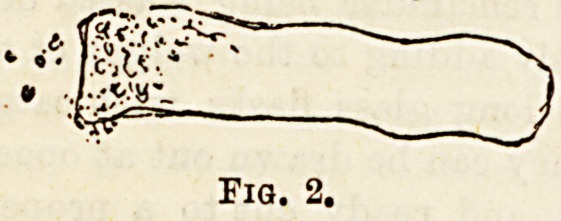


**Fig. 3. f2:**
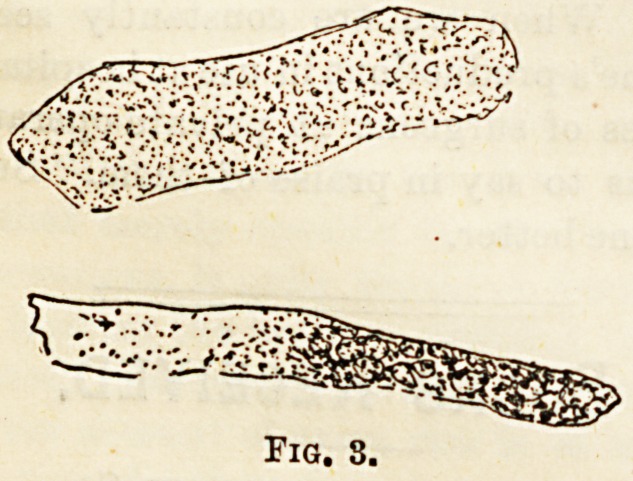


**Fig. 4. f3:**
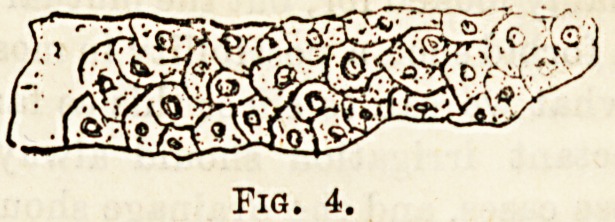


**Fig. 5. f4:**
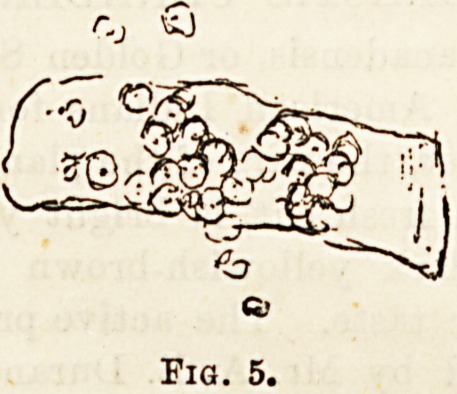


**Fig. 6. f5:**